# Immune modulation of gut microbiota and its metabolites in chronic hepatitis B

**DOI:** 10.3389/fmicb.2023.1285556

**Published:** 2023-11-29

**Authors:** Shi-Qin Li, Yue Shen, Jun Zhang, Cheng-Zhao Weng, Sheng-Di Wu, Wei Jiang

**Affiliations:** ^1^Department of Gastroenterology and Hepatology, Zhongshan Hospital (Xiamen), Fudan University, Xiamen, China; ^2^Department of Gastroenterology and Hepatology, Zhongshan Hospital, Fudan University, Shanghai, China; ^3^Shanghai Institute of Liver Diseases, Fudan University Shanghai Medical College, Shanghai, China

**Keywords:** gut microbiota, gut microbiota-derived metabolites, chronic hepatitis B, fecal bacteria transplantation, immune cells

## Abstract

The gut microbiota is a diverse ecosystem consisting of 100 trillion microbiomes. The interaction between the host’s gut and distal organs profoundly impacts various functions such as metabolism, immunity, neurology, and nutrition within the human body. The liver, as the primary immune organ, plays a crucial role in maintaining immune homeostasis by receiving a significant influx of gut-derived components and toxins. Perturbations in gut microbiota homeostasis have been linked to a range of liver diseases. The advancements in sequencing technologies, such as 16S rRNA and metagenomics, have opened up new avenues for comprehending the intricate physiological interplay between the liver and the intestine. Metabolites produced by the gut microbiota function as signaling molecules and substrates, influencing both pathological and physiological processes. Establishing a comprehensive host-bacterium-metabolism axis holds tremendous potential for investigating the mechanisms underlying liver diseases. In this review, we have provided a summary of the detrimental effects of the gut-liver axis in chronic liver diseases, primarily focusing on hepatitis B virus-related chronic liver diseases. Moreover, we have explored the potential mechanisms through which the gut microbiota and its derivatives interact with liver immunity, with implications for future clinical therapies.

## 1 Introduction

Chronic liver disease has been a major global public health concern for decades. Although positive interventions are continuously being carried out in many countries and regions, chronic liver disease still imposes a severe economic burden on the state and society. In China, viral hepatitis is the most important cause of chronic liver disease. With the development of social economy, the incidence of non-alcoholic fatty liver disease (NAFLD) and alcoholic fatty liver disease (AFLD) is increasing and have brought changes to disease spectrum in the pathogenesis of liver cirrhosis and liver cancer (Wong et al., 2019; Xiao et al., 2019). Liver-derived bile is secreted into the bile duct system, while a variety of gut-derived signals, including bacterial products, environmental toxins and food antigens, are received in the gut and pass through the biliary tract into the liver (Albillos et al., 2020). Based on the “gut-liver axis,” a growing number of studies have found a relationship between hepatic injury and the dysregulation of gut microbiota homeostasis. In addition, an extensive signaling network, mediated by microbiota-derived metabolism, continues to receive attention and provide gut-liver targets for disease therapy (Sung et al., 2016). In this review, we have firstly summarized the disorder of the gut-liver axis and the characteristics of microorganisms in hepatitis B virus-related chronic liver diseases (HBV-CLD). Furthermore, the mechanism of gut microbiota and its derivatives in regulating the immune environment have also been discussed.

## 2 Gut microbiota and the gut-hepatic axis

The gut microbiota is a diverse ecosystem of 100 trillion microorganisms, including bacteria, fungi, viruses (bacteriophages), and protozoa. Bacteria are the most abundant and studied microbes in the gut. It can be divided into four phyla: Bacteroidetes, Firmicutes, Proteobacteria, and Actinobacteria (Eckburg et al., 2005; Heintz-Buschart and Wilmes, 2018). With the development of sequencing technology, more and more non-culturable bacteria have been identified. Microbiota, microbial genome and colonization environment construct microbiome. The gut microbiome genome is more than 150 times larger than the human genome. The human intestinal microbiome is the largest and most diverse microbial community in human body (Lynch and Pedersen, 2016). The “gut-liver axis” has received more and more attention in recent years. In early embryology, the liver originates in the foregut and carries out extensive material exchange. The exchange passes through the biliary tract, portal vein, and systemic circulation, forming the interlacing complex metabolism, the immunity and the neuroendocrine network. The liver filters blood absorbed through the gut via the portal venous system. Liver colonized macrophages (Kupffer cells, KCs) purify toxins and pathogens within the portal circulation (Hu et al., 2020). Gut-derived biological signals, such as bacterial products, environmental toxins and food antigens could be recognized by primary presenting cells and activate adaptive immunity after passing through the intestinal tight junction structure. Under homeostatic conditions, tight junction structures of the gut are able to prevent harmful substances and pro-inflammatory molecules from the internal environment (Peterson and Artis, 2014). Whereas, the overgrowth of small intestinal bacteria will lead to the increase of endotoxin release, the destruction of intestinal mucosal barrier function, an increase of intestinal permeability and etc. This in turn causes sustained activation of the host immune response (Sung et al., 2016; Hu et al., 2020). The entry of inflammatory cells and chemokines into portal circulation can lead to the injury of hepatocytes and the activation of hepatic stellate cells, which leads to the transformation of the liver from self-limited homeostasis to pathological scar repair (Hammerich and Tacke, 2023).

## 3 Alteration of gut microbiota in patients with chronic hepatitis B virus (HBV) infection

With the continuous development of high-throughput sequencing technologies including 16S rRNA sequencing and metagenomic deep sequencing, the analysis of the structure and genome of the gut microbiota has become much faster compared to those via traditional isolation and culture techniques. Many studies have carried out descriptive analyses of the composition and diversity of gut bacteria in disease (Cryan et al., 2019; Gilbert and Lynch, 2019; Hills et al., 2019; Tanoue et al., 2019). Chronic HBV infection is the leading cause of liver injury and dysfunction in China. Numerous studies, based on descriptive analysis, have demonstrated a relationship between the intestinal microbiota and chronic HBV infection (Chen et al., 2011a; Lu et al., 2011; Zeng et al., 2020; Wang et al., 2021).

The natural immune responses after chronic HBV infection include four stages: immune tolerance, immune clearance, low replication, and inactive stage. These stages are characterized by a sequence of complex interactions between the virus and the host immune system, which determine the clinical manifestation and outcome of the disease (Tsai et al., 2018). Based on the different immunological and virological characteristics, HBV infection may have varying effects on the gut microbiota in each stage. Multiple cross-sectional studies have analyzed the changes of gut microflora in different stages of liver diseases, including chronic hepatitis B (CHB), liver cirrhosis and hepatocellular carcinoma (HCC) (Huang et al., 2020). In 2017, Wang et al. compared the gut microbiota of patients with CHB (Child-Pugh score < 9) to that of healthy controls (HCs). It was found that CHB patients had a significant increase in Veillonellaceae OTU, while the OTU of *Alistipes*, *Bacteroides*, *Parabacteroides* and *Ruminococcus* were decreased (Wang et al., 2017). These findings suggest that dysfunction of the gut microbiota may precede liver damage and metabolic changes caused by HBV infection. In a similar vein, research in 2020 analyzed the gut microbiota of patients with hepatitis B fibrosis (liver biopsy pathology grade F0-F4). The study observed a decrease in *Bacteroides* and *Ruminococcus*, whereas there was a significant increase in *Prevotella* abundance compared to HCs. As the pathological grade of liver fibrosis increased, the abundance of Bacteroidetes continued to decrease (Wang et al., 2020). Additionally, it was found that the abundance of butyrate-producing Anaerostipes was higher in patients who tested positive for HBsAg (a marker for HBV infection) and had normal levels of alanine transaminase (ALT) compared to HBsAg-negative controls. Within the HBsAg positive population, the abundance of *Megasphaera*, a genus belonging to the Firmicutes phylum, was higher in patients with elevated ALT levels than in those with normal ALT levels. Furthermore, the abundance of *Megasphaera* was positively correlated with ALT levels. These findings suggest that the replication of HBV can indeed influence the composition of the gut microbiota and liver metabolism (Yun et al., 2019). In addition, in 2020, Chen et al. reported on a cross-sectional cohort that included 3 groups of patients: hepatitis B carrier, CHB, and HBV-LC. The *α* diversity decreased significantly along with disease progression and was lowest among HBV-LC. The findings of Chen et al. contrasted with the results of another study by Zeng et al. (2020). In Zeng et al’s analysis at the phylum level, the B/F ratio gradually decreased from healthy individuals to cirrhotic patients, while the abundance of Proteobacteria and Actinobacteria increased as the disease advanced. At the genus level, Bacteroides levels decreased in all four groups, while the abundance of *Haemophilus*, *Fusobacterium*, *Veillonella*, *Streptococcus*, and *Ruminococcus* increased as the course of CHB progressed. Moreover, Chen et al. (2020) also highlighted two specific strains, *D. succinatiphilus* and *A. onderdonkiil*, of which the abundance exhibited sustained decrease along with liver damage aggravation. In our latest research, we compared the microbiota distribution and abundance between HCs and HBV-CLD, nine genera (*Blautia*, *Bifidobacterium*, *Escherichia-Shigella*, *Klebsiella*, *Parasutterella*, *E.hallii group*, *Collinsella*, *Erysipelotrichaceae_UCG-003*, *Lactococcus*) were found to be enriched in HC subjects, whereas six genera (*Faecalibacterium*, *Streptococcus*, *Sutterella*, *Lachnospiraceae_ND-3007*, *Ruminiclostridium 9*, *Lachnospiraceae_UCG-010*) were markedly increased in HBV-CLD patients (Table 1; Shen et al., 2023).

**Table 1:** Changes in gut microbiota during the course of chronic HBV infection.

	Health	Hepatitis	Liver cirrhosis
Increased	Blautia (Shen et al., 2023)	Veillonellaceae (Wang et al., 2017; Chen et al., 2020)	Proteobacteria (Chen et al., 2011b; Wei et al., 2013; Leclercq et al., 2014; La Rosa et al., 2019; Chen et al., 2020)
	Escherichia-Shigella (Shen et al., 2023)	Prevotella (Wang et al., 2020)	Fusobacteria (Chen et al., 2011b; Wei et al., 2013)
	Bifidobacterium (Shen et al., 2023)	Anaerostipes (Yun et al., 2019)	Enterobacteriaceae (Chen et al., 2011b)
	Klebsiella (Shen et al., 2023)	Megasphaera (Yun et al., 2019)	Streptococcaceae (Chen et al., 2011b, 2020)
	Parasutterella (Shen et al., 2023)	Faecalibacterium (Shen et al., 2023)	Veillonella (Wei et al., 2013; Chen et al., 2020)
	E. hallii (Shen et al., 2023)	Streptococcus (Shen et al., 2023)	Bacteroidetes/Firmicutes ratio (Leclercq et al., 2014; La Rosa et al., 2019)
	Collinsella (Shen et al., 2023)	Sutterella (Shen et al., 2023)	Dorea (Leclercq et al., 2014; La Rosa et al., 2019)
	Erysipelotrichaceae (Shen et al., 2023)	Lachnospiraceae (Shen et al., 2023)	Coprococcus (Pedersen et al., 2019)
	Lactococcus (Shen et al., 2023)	Ruminiclostridium (Shen et al., 2023)	Haemophilus (Chen et al., 2020)
			Fusobacterium (Chen et al., 2020)
			Streptococcus (Chen et al., 2020)
			Bifidobacterium dentium (Xu et al., 2012)
Decreased			
		Alistipes (Wang et al., 2017)	Bacteroidetes (Wang et al., 2020)
		Bacteroides (Wang et al., 2017, 2020)	Megamonas
		Parabacteroides (Wang et al., 2017)	Roseburia (Leclercq et al., 2014; La Rosa et al., 2019)
		Ruminococcus (Wang et al., 2017, 2020)	Succinatiphilus (Chen et al., 2020)
			Onderdonkiil (Chen et al., 2020)
			Bifidobacterium streptozotocin (Xu et al., 2012)
			Bifidobacterium Pseudostreptozotocin (Xu et al., 2012)

In patients with HBV-associated liver cirrhosis (HBV-LC), there was a decrease in the α diversity of the gut microbiota. The proportion of Bacteroidetes was significantly reduced and negatively correlated with the severity of liver disease as measured by the Child-Pugh grade. In contrast, there was an increased abundance of bacteria belonging to the phyla Proteobacteria and Fusobacteria in HBV-LC patients (Chen et al., 2011b; Wei et al., 2013). Specifically, the abundance of Enterobacteriaceae and Streptococcaceae families was significantly increased in patients with HBV-LC, with the most significant elevation observed in patients with sclerosing liver disease (Chen et al., 2011b). Furthermore, there was a marked elevation of the genus *Veillonella* in patients with HBV-LC (Wei et al., 2013). In 2011 Xu et al. discovered that the levels of Bifidobacterium streptozotocin and Bifidobacterium Pseudostreptozotocin were significantly lower in HBC-LCs than in HCs. However, the level of *Bifidobacterium dentium* was increased in patients compared with HCs (Table 1; Xu et al., 2012).

In 2019, Zeng et al. (2020) conducted an analysis of a cohort consisting of patients with CHB, HBV-LC, and HCC. However, when analyzing at the phylum level, they observed certain differences. The Bacteroidetes/Firmicutes (B/F) ratio was found to be increased in patients with CHB. The abundance of Proteobacteria, a phylum that includes various pathogenic bacteria, was also increased, particularly in patients with cirrhosis and HCC. The abundance of *Roseburia*, a bacterial genus known to promote the growth of other beneficial bacteria by degrading dietary β-mannan decreased in the progression of CHB. On the other hand, the pro-inflammatory bacterial genus *Dorea* showed an increased abundance (Leclercq et al., 2014; La Rosa et al., 2019). *Bifidobacterium* abundance decreased in all three stages (CHB, HBV-LC, and HCC) compared to healthy individuals, while the drop was least in patients with HCC. The abundance of *Coprococcus,* a pro-inflammatory bacterium associated with high-fat diet, was significantly increased in CHB and HBV-LC stages, whereas it was significantly decreased in HCC patients (Table 1; Pedersen et al., 2019; Zeng et al., 2020). These results suggest that in progression of CHB, there are massive and complex changes in the gut microbiota.

These results contribute to our understanding of the altered microbiota composition in HBV-CLD. The differential abundance of specific genera between HCs and HBV-CLD patients suggests potential microbial markers and highlights the potential role of these genera in the pathogenesis and progression of the disease. However, further research is necessary to elucidate the functional implications of these findings and their potential clinical significance in the context of HBV-CLD. In addition to studying changes in the structure and quantity of gut microbiota, the identification of specific microorganisms is crucial for determining the disease-causing pathogens of diseases with unclear pathogenic mechanism. Detecting and understanding the involvement of particular pathogens is essential for determining their role as disease-causing agents (Table 2).

**Table 2:** Primary study of gut microbiome in HBV-related liver disease.

Reference of the study	Year	Sample size	Sample type	Methodology	Key findings
Huang et al. (2020)	2023	HBV-related HCC patients (*n* = 113) Healthy controls (*n* = 100)	Fecal	16S rRNA sequencing	(1) Changes caused by the gut microbiota via serum bile acids may be important factors associated with HCC burden and adverse clinical outcome. (2) Gut microbes can be used as biomarkers of clinical features and outcomes.
Shen et al. (2023)	2023	HBV-CLD patients (*n* = 64) Healthy controls (*n* = 17)	Fecal and blood	16S rRNA sequencing	(1) CHB progression and antiviral treatment are two main factors contributing to the compositional shift in microbiome and metabolome of HBV-CLD patients. (2) Peripheral immunity might be an intermediate link in gut microbe-host interplay underlying CHB pathogenesis.
Wang et al. (2021)	2021	HBV-ACLF (*n* = 212) CHB patients (*n* = 252) HBV-associated cirrhosis (*n* = 162) Healthy controls (*n* = 877)	Fecal	16S rRNA sequencing	(1) High abundance of Enterococcus is associated with progression while that of *Faecalibacterium* is associated with regression of HBV-acute-on-chronic liver failure. (2) The microbiota features hold promising potential as prognostic markers for HBV-acute-on-chronic liver failure.
Chen et al. (2020)	2020	CHB patients (*n* = 76) Healthy controls (*n* = 21)	Fecal	16S rRNA sequencing	(1) The compositional and network changes in the gut microbiota in varied CHB stages, suggest the potential contributions of gut microbiota in CHB disease progression.
Yun et al. (2019)	2019	HBsAg-positive (*n* = 36) Healthy controls (*n* = 76)	Fecal and blood	16S rRNA sequencing	(2) The replication of HBV can indeed influence the composition of the gut microbiota and liver metabolism.
Wang et al. (2017)	2017	CHB patients (*n* = 85) Healthy controls (*n* = 22)	Fecal and blood	Illumina MiSeq sequencing platform and gas chromatography mass spectrometry	(1) Dysfunction of the gut microbiota may precede liver damage and metabolic changes caused by HBV infection.
Xu et al. (2012)	2012	CHB patients (*n* = 16) HBV-related cirrhosis (*n* = 16) Healthy controls (*n* = 15)	Fecal	Nested-PCR-based denaturing gradient gel electrophoresis (PCR-DGGE), clone library, and real-time quantitative PCR	(1) The composition of intestinal Bifidobacterium was deeply altered in CHB and HBV cirrhotic patients with a shift from beneficial species to opportunistic pathogens. (2) The dysbiosis of the intestinal microbiota in patients with HBV-induced chronic liver disease potentially serve as guidance for the probiotic interventions of these diseases.
Chen et al. (2011b)	2011	HBV-related cirrhosis (*n* = 36) Healthy controls (*n* = 24)	Fecal	16S rRNA sequencing	(1) Fecal microbial communities are distinct in patients with cirrhosis compared with healthy controls. (2) The prevalence of potentially pathogenic bacteria, such as Enterobacteriaceae and Streptococcaceae, with the reduction of beneficial populations such as Lachnospiraceae in patients with cirrhosis may affect prognosis.

## 4 The interaction between gut microbiota and anti-viral treatment of hepatitis B

### 4.1 The influence of gut microbiota on anti-virus immunity of hepatitis B

Numerous studies have demonstrated that the gut microbiota plays vital role in influencing the immune response of the host after HBV infection. The production of interferon, proinflammatory cytokine and the recruitment of immune cells to suppress viral replication are the basis of liver inflammation and injury (Ma et al., 2014). In adults, approximately 5% of acute HBV infections progress to CHB. However, the risk of chronic infection is significantly higher in newborns, with more than 90% of them developing CHB if being infected perinatally. Additionally, in children aged 1–5 years, the risk of chronic infection ranges from 30 to 50% (Nicholson et al., 2012b). The maturation of the immune system is now recognized as a significant factor influencing the persistence of HBV infection. It has been reported that the stability of the gut microbiota appears to play a pivotal role in the age-dependent clearance of HBV in serology. Chou et al. conducted a study in an animal model that demonstrated the involvement of the gut microbiota in the clearance of HBV virus. Adult mice with mature and stable intestine were able to clear the HBV virus 6 weeks after infection. Young mice lacking a mature and stable gut microbiota showed persistent viral infection. Oral administration of antibiotics for a duration of 6–12 weeks blocked the clearance of HBV in adult mice (Chou et al., 2015; Guo et al., 2021). In another study conducted in 2022, Wang et al. depleted the gut microbiota in BALB/c mice using broad-spectrum antibiotics (ABX) and then reconstituted it with fecal microbiota from either naïve BALB/c or C57BL/6 mice. The results showed that HBV infection was transient in BALB/c mice, but the fecal microbiota from C57BL/6 mice induced immune tolerance in the liver, prolonging HBV infection. The study concluded that the outcomes of HBV infection in mice are determined by both the host’s genetic background and the composition of the gut microbiota. It was also observed that commensal bacteria from fecal microbiota transplantation (FMT) partially, but selectively, colonize the gut of the new host. FMT has the potential to modulate the host’s immune response and alter its susceptibility to HBV infection (Wang et al., 2022).

### 4.2 The effect of anti-HBV therapy on gut microbiota

A 2020 study evaluated changes in gut microbiota composition before and after entecavir treatment in a mouse with persistent HBV infection mediated by recombinant AAV8 virus (rAAV8-HBV). The results demonstrated that rAAV8-HBV-infected mice exhibited a decrease in α diversity of gut microbiota, which significantly recovered after 4 weeks of ETV treatment. In the HBV-infected mouse model, the abundance of Blautia Brucella and Clostridium was significantly reduced, and these reductions were negatively correlated with the levels of HBsAg and HBeAg. On the other hand, the groups of Butyricicoccus and Prevotellaceae showed a positive association with HBsAg and HBeAg levels. Interestingly, the gut barrier-protecting bacterium Akkermansia was found to be significantly reduced in HBV-infected mice. However, treatment with ETV restored its levels to be equivalent to those observed in HCs (Plovier et al., 2017; Li et al., 2020). In addition, it was reported that the relative abundance of Blautia, Dorea, and Ruminococcaceae_UCG-013 was similar between HCs and CHB patients who received antiviral treatment compared to those without treatment, indicating that antiviral treatment may help rebuild intestinal homeostasis in CHB patients (Shen et al., 2023). Considering that chronic hepatitis C virus (HCV) infection can be cured with antiviral therapy and achieving sustained virological response (SVR), it serves as a more ideal model for investigating the impact of liver disease on the gut microbiota compared to HBV infection. In 2021, Freya et al. prospectively analyzed alterations in the gut microbiota of CHC patients who achieved an antiviral drug and acquired SVR. Over the course of SVR24/48 weeks, notable statistical differences were observed in α diversity and the overall microbial community, specifically in patients without cirrhosis. These differences included a significant increase in the abundance of Collinsella and Bifidobacterium (Wellhoner et al., 2021). It can be concluded that antiviral treatment in CHC patients leads to the alteration of gut microbiota, which would differ as the fibrotic stages. This observation indicates that antiviral treatment in viral hepatitis plays an important role in influencing the composition of the gut microbiota.

## 5 Advances in gut microbiota-related metabolites in chronic liver disease

Characterization of complex microbial communities using 16S rRNA sequencing lacks quantitative functional annotation. However, this limitation can be overcome by analyzing microbial-derived metabolites through the fecal metabolome. The study of gut microbiota metabolites as intermediaries that influence the host is gaining more attention (Shi et al., 2016; Sun et al., 2021). Gut microbiota-derived metabolites are able to shape and regulate host immune system. Increasing evidence indicates that these metabolites, such as trimethylamine, bile acid, short-chain fatty acid and ethanol, play an important role in the initiation and progression of disease (Chu et al., 2019; Pant et al., 2019; Zhao et al., 2020). Therefore, the systematic integration of the gut microbiome and metabolome holds the potential to reveal previously undiscovered information about the gut-liver axis. Further understanding of the variations in gut microbiota during different physiological pathology conditions can be utilized for the diagnosis and intervention of related diseases, paving the way for new insights and approaches to diagnosis and intervention of related diseases.

### 5.1 Short-chain fatty acid

**Figure 1:**
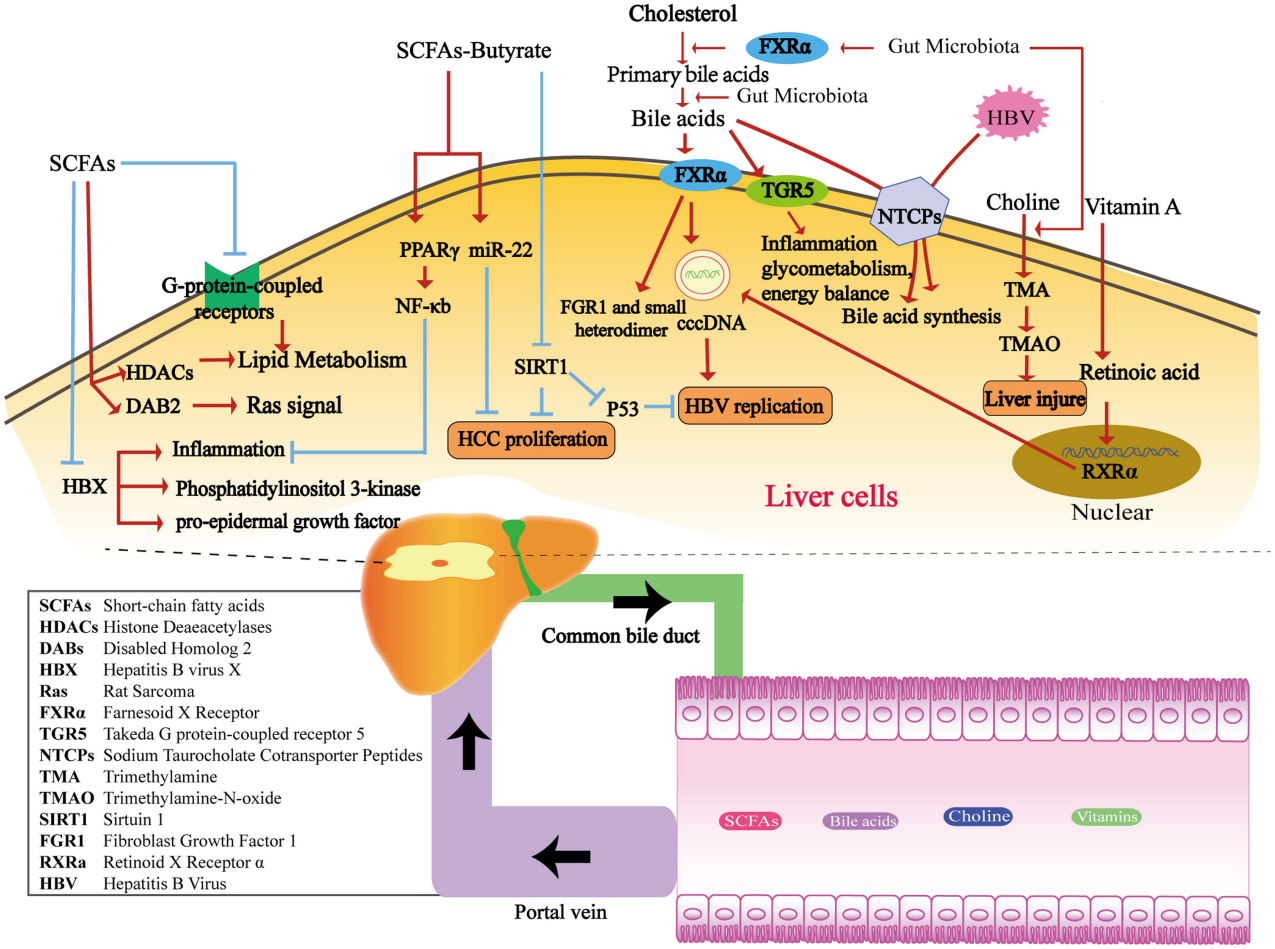
The metabolism of gut microbiota-derived compounds passes through the portal vein into the liver, affecting HBV replication and HCC proliferation. SCFAs produced by the intestinal flora play a crucial role in inflammation and several signaling pathways, including phosphatidylinositol 3-kinase, pro-epidermal growth factor, and Ras-related pathways. They achieve this by regulating HBX, HDACs, DAB2, and G-protein-coupled receptors (Koh et al., 2016). Butyrate inhibits inflammation in the liver by stimulating PPARγ and downstream NF-κB signaling pathways (Yang et al., 2018). Additionally, it plays a crucial role in suppressing HCC proliferation by activating miR-22 or inhibiting SIRT1 (Pant et al., 2017). Moreover, the SIRT/p53 pathway can also impede HBV-DNA replication under the stimulation of butyrate (Pant et al., 2019). Enzymes from various gut microbiota transform primary bile acids into secondary bile acids. FXRα and TGR5 are both important receptors for BAs. The gut microbiota affects the synthesis of primary bile acids through the FXRα pathway. FXRα stimulates HBV transcription by binding to cccDNA, and downstream targets include FGR1 and the small heterodimer partner. TGR5 plays a role in regulating inflammatory diseases, glycometabolism, and energy balance (Radreau et al., 2016; Jia et al., 2018). During HBV infection, HBV competes with BAs to bind with NTCPs and suppresses BA intake. This reduction in NTCP functionality contributes to the elevated levels of BAs observed during HBV infection (Oehler et al., 2014). The gut microbiota has the ability to convert dietary choline into TMA, which is then converted to TMAO by the liver through the action of flavin monooxygenase. Elevated levels of TMAO have been observed in rats with liver lesions (Janeiro et al., 2018). Retinoic acid can enhance HBV transcription and replication by activating the RXRa in nuclear receptors (Huan and Siddiqui, 1992; Huan et al., 1995).

Short-chain fatty acids (SCFAs) are saturated fatty acids containing one to six carbon atoms, including acetate, propionate and butyrate. SCFAs are primarily produced through the fermentation of indigestible polysaccharides within the colon. This fermentation process is carried out by various bacterial phyla, including Actinobacteria, Bacteroidetes, Firmicutes, and Proteobacteria (Oliphant and Allen-Vercoe, 2019). For example, the main producers of butyrate are Clostridia, Eubacteria, and Roseburia microbes (Nicholson et al., 2012a). SCFAs regulate liver immune homeostasis and lipid metabolism by activating G-protein-coupled receptors or inhibiting the activity of histone deacetylase (HDAC) (Koh et al., 2016). In 2021, McBrearty et al. conducted a study in which they investigated the effects of short-chain fatty acid (SCFA) supplementation, consisting of the sodium salts of butyrate, propionate, and acetate, in Hepatitis B virus X (HBx) transgenic mice aged 9–12 months. The results of the study showed that the SCFA supplementation reduced the occurrence of liver dysplasia and liver tumors in these mice. Furthermore, the researchers found that SCFAs inhibited the inflammation, phosphatidylinositol 3-kinase, pro-epidermal growth factor, and Ras-related signaling pathways mediated by HBX. Additionally, SCFA supplementation up-regulated the expression of the cancer suppressor factor, disabled homolog 2 (DAB2) (Figure 1; McBrearty et al., 2021). These findings suggest that SCFAs may involve in the mechanism of delaying the progression of chronic liver disease and the development of HCC.

Butyrate is one of the most widely studied short-chain fatty acid. It plays an important role in the regulation of intestinal barrier function, intestinal immunity, and inflammation response. It promotes the secretion of glucagon-like peptide by activating free fatty acid receptor-3, which subsequently inhibits food intake and gastric emptying. Meanwhile, butyrate can bind to and activate peroxisome proliferator-activated receptor γ(PPARγ), thereby exerting an anti-inflammatory effect through the antagonism of nuclear factor-κB transcription (Cani et al., 2019). Butyric acid produced by the gut microbiota has been shown to impact the development of liver disease through multiple mechanisms. The abundance of butyrate-producing bacteria in the faeces of patients with HBV-LC and HCC is significantly decreased (Ren et al., 2019; Zeng et al., 2020). Hepatic histone deacetylation Sirtuin-1 (SIRT-1), as a target molecule of butyric acid, has emerged as a key focus in understanding the mechanism. In the liver of CHB patients with high viral replication and in HBV-positive HCC cells, Butyrate has been reported to inhibit the proliferation and induce apoptosis. This effect was achieved by suppressing the expression of SIRT-1 and simultaneously up-regulating the expression of miR-22 (Pant et al., 2017). It is also reported that butyrate can inhibit HBV replication in HEPG2.2.15 cells by regulating the SIRT-1/P53 pathway (Figure 1; Pant et al., 2019).

### 5.2 Bile acids

Bile acids (BAs) are synthesized from cholesterol in the liver and stored in the gallbladder before being released into the intestine. Primary bile acids, such as cholic acid and deoxycholic acid, are transformed by bile salt metabolism enzymes produced by various gut microbiota. These enzymes facilitate binding, dehydrogenation, and dehydroxylation processes, leading to the production of over 50 different secondary bile acids, including deoxycholic acid, lithocholic acid, and ursodeoxycholic acid. Certain bacteria, such as Clostridium, Bacteroides, Lactobacillus, Bifidobacterium, and Enterococcus, play a role in the conversion of primary bile acids through the production of bile salt hydrolase. Additionally, anaerobic bacteria like *Clostridium* sp. contribute to further modifications through a process called 7α/7β dehydroxylation (Long et al., 2017; Jia et al., 2018). The gut microbiota exerts a notable influence on the synthesis of primary bile acids in the liver via the Farnesoid X receptor (FXR) pathway. Fibroblast Growth Factor 1 (FGR1) and the small heterodimer partner are downstream targets within this pathway (Inagaki et al., 2006; Sayin et al., 2013). In addition to FXR, Takeda-g-protein-receptor-5 (TGR5) represents another well-known bile acid receptor. TGR5 plays a role in regulating inflammatory diseases, glycometabolism, and energy balance (Figure 1; Jia et al., 2018).

HBV infection has an important effect on bile acid metabolism. Sodium taurocholate cotransporter peptides (NTCPs) in the hepatocyte basement membrane mediate hepatic uptake of bile salts from portal vein blood (Yan et al., 2012). Furthermore, the NTCPs also serve as receptors for HBV invasion into hepatocytes through their binding to the pre-S1 domain of the HBV virus (Konig et al., 2014). The competitive binding of HBV to NTCPs leads to a reduction in salt uptake and a compensatory increase in bile acid synthesis within hepatocytes. In human liver chimeric mice infected with HBV, elevated activity of cholesterol 7α-hydroxylase is observed, along with the downregulation of FXR and downstream small heterodimer partner expression. Additionally, HBV-induced hepatocyte injury results in a decrease in the number of NTCPs, leading to impaired uptake and transformation capacity of bile acids. This reduction in functionality of NTCPs contributes to the elevated levels of bile acids observed during HBV infection (Oehler et al., 2014). The levels of total bile acids and primary bile acids are elevated in patients with HBV-related liver cirrhosis (HBV-LC), whereas the total bile acids and secondary bile acids in feces exhibit a significant decrease. In patients diagnosed with HBV-LC, dynamic changes in bile acid profiles are indicative of Child-Pugh grades, reflecting the progression of liver function deterioration (Wang et al., 2016). Bile acid metabolism also has an impact on viral replication, especially in the context of HBV infection. Both FXRα and BAs play crucial roles in HBV replication, making FXRα a potential therapeutic target for HBV treatment. Activated by BAs, FXRα stimulates HBV transcription by binding to the core promoter and enhancer regions of HBV covalently closed circular DNA (cccDNA). Studies have demonstrated that the reduction of FXR expression using an FXR agonist effectively lowers FXR expression in HBV-infected cell lines. This reduction in FXR expression has been shown to decrease the levels of PgRNA, PreC-RNA, rcDNA, cccDNA, HBsAg, and HBeAg, which are all important markers of HBV replication and viral activity (Figure 1; Radreau et al., 2016). Clinical trials are currently underway to evaluate the potential clinical application of new FXR agonists, including EYP001 and ASC42 in combination with pegylated interferon and nucleotide analogues.

### 5.3 Choline

Choline, an essential nutrient and phospholipid constituent of cell membranes, exerts a significant impact on liver function. Choline deficiency affects the liver through several mechanisms, such as impaired formation of very low-density lipoproteins, mitochondrial dysfunction, and endoplasmic reticulum (ER) stress (Zeisel and da Costa, 2009). Dietary choline derived from sources such as eggs, milk, and red meat is metabolized by the gut microbiota, leading to the production of trimethylamine (TMA). This microbial process reduces the bioavailability of choline. Subsequently, the liver converts TMA to trimethylamine-n-oxide (TMAO) through the action of flavin monooxygenase. Bacteria have the ability to compete with the host for choline, which can influence the metabolism of methyl donors in the plasma and liver. Consequently, these interactions can result in alterations to host epigenetics and heightened susceptibility to metabolic diseases (Figure 1; Janeiro et al., 2018). Based on current research, there is evidence linking the production of trimethylamine (TMA) and trimethylamine-n-oxide (TMAO) through choline metabolism to various diseases, such as cardiovascular disease, diabetes, and chronic kidney disease (Zeisel and Warrier, 2017; Wang and Zhao, 2018). TMAO can further exacerbate impaired glucose tolerance, promote the expression of proinflammatory factors like MCP-1, and diminish the levels of the anti-inflammatory cytokine IL-10 in adipose tissue (Figure 1; Gao et al., 2014). A study reported that hepatic steatosis was found to be associated with the metabolite N, N, N-trimethyl-5-aminolevulinic acid (TMAVA), which is produced through the metabolism of trimethyl lysine by *Enterococcus faecalis* and *Pseudomonas aeruginosa* (Zhao et al., 2020). Plasma levels of TMAVA were observed to be significantly higher in patients with hepatic steatosis compared to HCs. Additionally, in a hepatotoxicity test involving Bay41-4109, a novel anti-HBV compound, elevated levels of TMAO were found in the group of rats with liver lesions induced by Bay41-4109 (Shi et al., 2007). The effects of TMAO in HBV-CLD still require further exploration. Cox et al. conducted an analysis using urine magnetic resonance spectroscopy in patients with CHB, HBV-LC, HCC, as well as HCs. The results suggested that TMAO is among the major metabolites that contribute to distinguishing the three disease stages. Furthermore, the significant decrease in TMAO level observed in patients with HCC suggests its potential utility as a diagnostic biomarker (Cox et al., 2016).

### 5.4 Vitamins

Vitamins serve as cofactors for numerous metabolic enzymes that play a critical role in maintaining normal cell function, growth, and development. In addition, the homeostasis of gut microbiota is influenced by interactions with vitamin synthesis. Host-expressed nuclear receptors, such as the receptors for vitamin A and vitamin D, act in synergy on intestinal epithelial and mucosal immunity, contributing to the maintenance of gut microbiota homeostasis (Cantorna et al., 2019). Vitamin A and D deficiency leads to decreased gut microbial diversity, imbalance of the microbiota, and increases susceptibility of the organism to gastrointestinal infections or injury. On the other hand, the gut microbiota is also able to synthesize B and K vitamins (Magnusdottir et al., 2015; Karl et al., 2017). Deficiency in vitamins A and D can result in reduced gut microbial diversity, microbiota imbalance, and increased vulnerability to gastrointestinal infections or injuries. Conversely, the gut microbiota has the capability to synthesize vitamins B and K (Dowling and Wald, 1960). Studies have shown that retinoic acid can enhance HBV transcription and replication by activating retinoid X receptor α (RXRα) (Figure 1; Huan and Siddiqui, 1992; Huan et al., 1995). HBV infection could, in turn, negatively impact retinol metabolism and promote the expression of related proteins, including RNA binding protein (RBP), cellular retinol-binding proteins (CRBP1), and Aldehydedehydrogenase1 (ALDH1) (Mawson and Steele, 2001). Hence, HBV infection can be facilitated by enhancing the transport of retinol into cells. The conversion of retinol to retinoic acid activates RXR, leading to the promotion of viral replication. The active form of vitamin D is known as 1,25(OH)_2_D3, while 25 (OH)D3 is more stable and easier to measure. In patients with active CHB, there is a decrease in serum 25(OH)D3 levels. The level of 25(OH)D3 is associated with HBV-DNA load. However, the precise molecular mechanism of how 25(OH)D3 functions in CHB is yet to be fully understood (Farnik et al., 2013).

## 6 Gut microbiota and liver immunity

Maintaining a delicate equilibrium between immune activation and tolerance is of utmost importance for the liver, as it continually receives diverse signals from the gut via the biliary tract, portal vein, and systemic circulation. Emerging research has revealed that the composition of the gut microbiota plays a pivotal role in shaping the development of the immune system and influencing the functional diversity of immune cells. Consequently, we present a comprehensive overview of the interaction networks involving the intestinal microbiota, microbiota-derived metabolites, immune system of liver and HBV.

### 6.1 Macrophages

**Figure 2:**
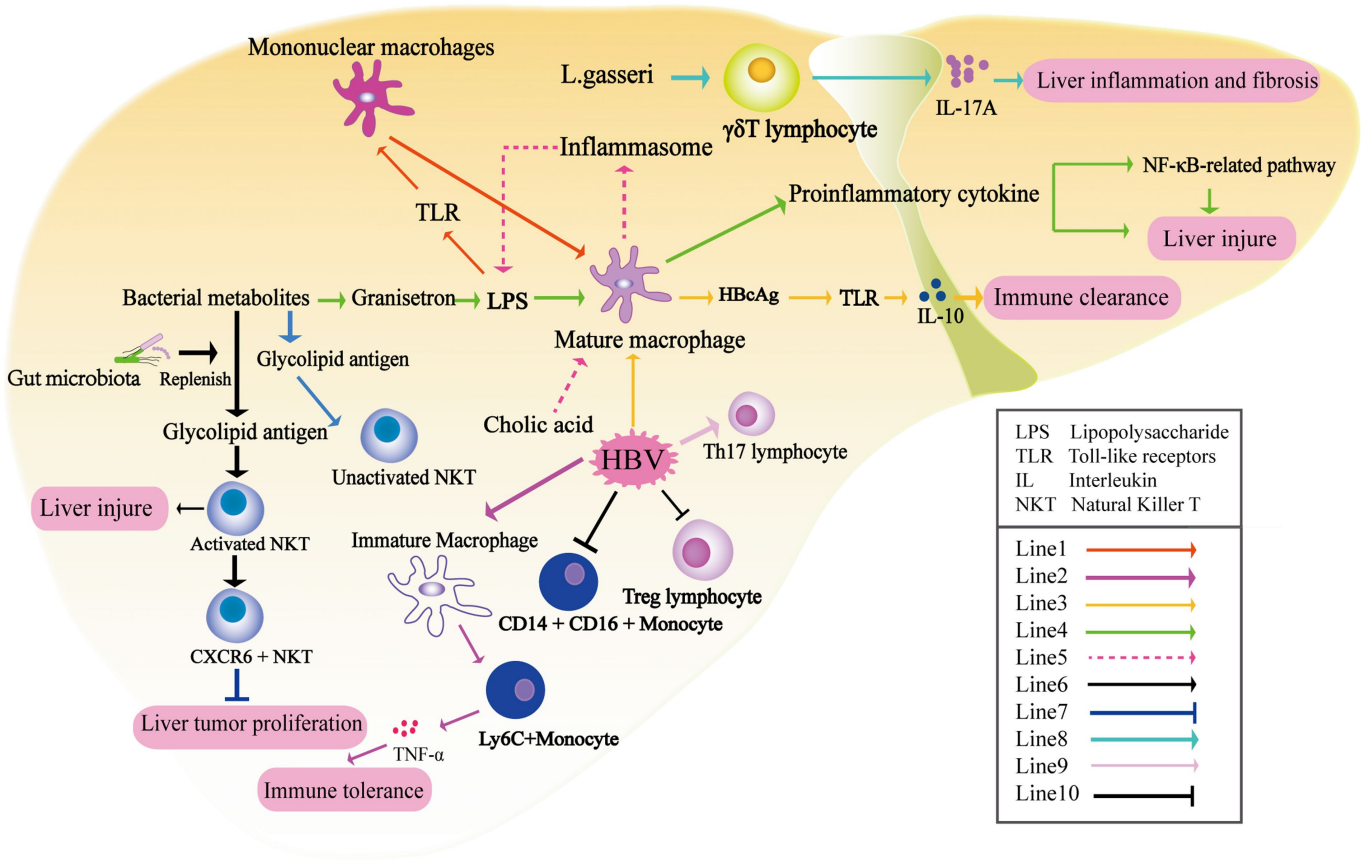
The liver undergoes immune modulation under the influence of metabolites produced by the gut microbiota. Prolonged exposure to LPS leads to macrophage tolerance and downregulation of TLR expression (Line1) (Rivera et al., 2007). The maturation of KCs plays a crucial role in age-dependent HBV clearance. Additionally, after HBV infection, there is a significant increase in the number of Ly6C+ monocytes secreting TNF-α in the liver, which mediates the immune tolerance of CHB (Line2) (Wu L. L. et al., 2019). HBcAg activates TLR2 on KCs, resulting in the production of IL-10 (Line3) (Li et al., 2015). The microbiota-derived 5-HT3 antagonist granisetron inhibits the production of proinflammatory cytokines by macrophages following LPS stimulation (Gong et al., 2019). Recognition between TLR4, LPS, and LPS-binding protein triggers the activation of KCs, leading to the activation of the NF-κB-related pathway and subsequent production of inflammatory cytokines, thus inducing acute liver injury (Line4) (Yang et al., 2018). Cholic acid up-regulates the inflammasome in macrophages, promoting the onset and progression of cholestatic liver disease (Line5). The activation of natural killer T (NKT) cells in the liver is reliant on the presence of the gut microbiota (Line6) (Chen et al., 2014; Liang et al., 2014). CXCR6+ NKT cells recruited to the liver can inhibit liver tumor growth when the level of CXCL16 increases due to the application of antibiotics specifically targeting gram-negative bacteria (Line7) (Ma et al., 2018). *Lactobacillus gasseri* exacerbates liver inflammation and fibrosis by inducing the production of IL-17 by γδT cells (Line8). HBV has the ability to expand Th17 lymphocytes (Line9), increase intermediate monocytes (CD14 + CD16+), and suppress regulatory Treg lymphocytes under the influence of gut metabolites (Line9) (Shen et al., 2023).

Macrophages constitute a crucial component of the innate immune system. In the liver, macrophages encompass both monocyte-derived macrophages in the periphery and resident Kupffer cells (KCs), which account for approximately 80–90% of the total resident macrophages *in vivo*. These macrophages play a pivotal role in regulating liver immune homeostasis through functions such as phagocytosis and antigen presentation. Toll-like receptors (TLRs) present in KCs can be activated by various endogenous and exogenous stimuli, including endotoxin. Specifically, the Lipopolysaccharide-TLR axis (particularly TLR-4) plays a significant role in the hyperactivation of macrophages in NAFLD. In the physiological state, sustained exposure to lipopolysaccharide (LPS) leads to macrophage tolerance and the downregulation of TLR expression. However, under pathological conditions such as lipotoxicity in NAFLD, the sensitivity of hepatic macrophages to LPS continues to increase (Rivera et al., 2007; Vespasiani-Gentilucci et al., 2015). TLR4, presented on the surface of mononuclear macrophages, is capable of recognizing and binding to the complex formed by LPS and LPS-binding protein. This interaction triggers the activation of CD14+ KCs, leading to the activation of the NF-κB-related pathway and subsequent production of inflammatory cytokines. Consequently, this process can induce acute liver injury (Yang et al., 2018). Research exploring the interaction between intestinal microflora and macrophages in HBV infection has indicated that the composition of the intestinal microbiota can impact the maturation of KCs. It has been observed that KCs derived from the livers of germ-free mice display an immune tolerance phenotype. The abundance of gut-derived microbial-associated molecular patterns is directly associated with the number, functional activity, and maturation status of KCs (Corbitt et al., 2013). It is reported that maturation of KCs in both transgenic and HBV-transfected mouse models is important regulatory factor of age-dependent HBV clearance. Wu et al. found that compared with 6-week-old C3H/HeN mice that developed immune tolerance after HBV transfection, the number of Ly6C+ monocytes secreting TNF-α in the liver was significantly increased, while the number of KCs secreting IL-10 was decreased in 12-week-old mice. LY6C+ monocytes and KCs represent the host defense mechanisms of immunity and tolerance, respectively, and play a crucial role in both HBV clearance and tolerance (Wu L. L. et al., 2019). Li et al. (2015) found that HBcAg promoted Il-10 by activating TLR2 on KCs after HBV infection (Figure 2). These results indicate the crucial role of KCs as an innate immunomodulatory center that regulates the maturation of the intestinal flora and is associated with the immune response outcomes in HBV infection. In addition, some microbiota-derived metabolites can also regulate the immune status of KCs. For instance, the gut microbiota-derived 5-HT3 antagonist granisetron has been shown to inhibit the production of proinflammatory cytokines by macrophages following LPS stimulation, thereby alleviating liver damage in septic mice (Figure 2; Gong et al., 2019). Chenodeoxycholic acid and deoxycholic acid have been shown to up-regulate the inflammasome in macrophages, thereby promoting the onset and progression of cholestatic liver disease. Following the up-regulation of inflammasomes in hepatic macrophages, there was an increase in intestinal permeability and alterations in the intestinal microbiota, thus further exacerbating the influx of endotoxins into the liver and aggravating cholestatic liver injury (Isaacs-Ten et al., 2020).

### 6.2 Natural killer T (NKT) cells

Animal experiments have shown that gut microbiota-derived antigens directly affect the composition and activation of NKT cells (Chen et al., 2014; Liang et al., 2014). NKT cells are comprised of two subsets: type I iNKT cells and type II NKT cells. These cells have the ability to recognize lipid antigens presented by the type I major histocompatibility complex (MHC)-like molecule CD1d. Through the secretion of cytokines, NKT cells can initiate specific adaptive immunity (Marrero et al., 2018). Glycolipid antigens derived from gut symbionts are important for NKT cells in concanavalin-induced liver injury. In the absence of gut microbiota, as observed in sterile mice, NKT cells are unable to be activated, resulting in liver damage in this model. These findings provide a mechanistic explanation for the gut microbiota’s ability to regulate liver inflammation (Wei et al., 2016). Furthermore, in HCC-related studies, antibiotics targeting gram-negative bacteria have also been found to balance the ratio of primary/secondary bile acids, which in turn increased the level of CXCL16 in sinusoidal endothelial cells. CXCR6+ NKT cells recruited to the liver can suppress liver tumor growth (Ma et al., 2018). In terms of metabolites, deficiencies in choline and phosphatidylcholine can impair the secretion of very low-density lipoprotein, leading to fat accumulation in the liver. Long-term feeding of a high-fat diet deficient in choline has been found to promote the occurrence and development of nonalcoholic steatohepatitis (NASH) and NASH-related HCC by stimulating iNKT cell activation, CD8+ T cell infiltration in the liver, and upregulation of inflammatory cytokines (Figure 2; Sutti et al., 2014). In conclusion, the gut microbiota and its associated metabolites play a significant role in the regulation of liver inflammation and injury through its impact on NKT cells.

### 6.3 γδt cells

The γδ lymphocyte (γδt) cells are abundant in the liver. Liver-resident γδt cells are the major producers of IL-17A. Importantly, the expansion of IL-17+ γδT (γδT-17) cells in response to inflammatory signals is crucial for the T-cell receptor (TCR) -mediated recognition of invading bacteria (Kumar et al., 2021). The presence of gut microbiota is crucial for maintaining the homeostasis of liver γδT-17 cells, encompassing their activation, survival, and proliferation. Furthermore, the quantity of microbiota directly impacts the population of γδT-17 cells in the liver (Li F. et al., 2017). *E. coli* has been reported to promote the production of γδT-17 cells in a dose-dependent manner. γδT cells have been shown to be involved in occurrence of liver diseases including NAFLD and primary sclerosing cholangitis (PSC) in response to gut microbiota-derived signals (Li F. et al., 2017; Tedesco et al., 2018; Torres-Hernandez et al., 2020). In a PSC-like mouse model, characterized by deficiency of the bile transporter multidrug resistance protein 2 (MDR2), increased intestinal permeability is observed. Furthermore, there is an elevated abundance of Lactobacillus in the feces, along with *Lactobacillus grigneri* specifically found in the liver. Liver MDR2^−/-^γδTCR^+^ cells exacerbate liver inflammation and fibrosis through the production of IL-17 upon stimulation by *L. gasseri* (Figure 2; Tedesco et al., 2018). Although γδT cells have been shown to fulfill various functions at different stages of immune responses during HBV infection, direct evidence regarding the interaction between HBV infection, gut microbiota, and γδT cells still lacks (Wu et al., 2013; Li Y. et al., 2017).

### 6.4 Lymphocytes and monocytes

The depletion of gut microbiota after antibiotic treatment was found to have detrimental effects on intestinal barrier function and to suppress T cell-mediated antiviral immune response in the liver. In mice subjected to HBV transfection, sustained high levels of serum HBsAg and negative HBsAb were observed at 6 weeks after antibiotic treatment, suggesting a potential influence of the gut microbiota on CD4+ T cell function. The reduced numbers of CD4+ T cells (CD44^hi^CD62L^−^CD4^+^ T cells) and their impaired ability to synthesize IFN-γ result in decreased proliferation of B cells and CD4+ follicular helper T cells within the germinal center of hair follicles. Consequently, the production of HBsAb was inhibited. Interestingly, it has been found that the restoration of anti-HBV immunity in mice requires a combination of antibiotics rather than a single type, highlighting the critical role of the overall gut microbiota in regulating anti-HBV immunity (Wu T. et al., 2019). In an HBV-infected mouse model, depletion of the gut microbiota induced by antibiotic treatment was associated with compromised integrity of the colonic epithelium. Additionally, the transfer of viable commensal gut microbiota from the small intestine to the liver was observed. Furthermore, the impaired function of T cells in antiviral responses within the liver was partially dependent on the upregulation of PD-1 expression (Guo et al., 2021). In HBV-CLD patient, the stimulation of bacterial extracts (BE) resulted in a decrease in pro-inflammatory intermediate monocytes (CD14 + CD16+), while classical monocytes (CD14 + CD16-) increased. Furthermore, BE derived from non-cirrhotic patients exhibited a significant enhancement in the expansion of T helper 17 lymphocytes (Th17) and suppressed regulatory T cell (Treg) lymphocytes (Figure 2; Shen et al., 2023). Except for viral hepatitis, transplantation of gut microbiota from NAFLD patients into recipient mice was also found to promote disease progression by recruiting and activating B cells within the liver. This observation suggests a potential pathogenic role of gut microbiota in driving B cell-mediated pathogenesis in NASH disease models (Barrow et al., 2021). Additionally, a recent Australian study conducted an experiment where peripheral blood mononuclear cells (PBMCs) from healthy individuals were stimulated with fecal microbiota extracts taken from patients with NAFLD, NASH, and NASH-related HCC. The researchers discovered that BE derived from the microbiota of NAFLD-HCC patients induced an immunosuppressive phenotype in T cells, as compared to the control group. This phenotype was characterized by an expansion of Treg cells and a reduction in CD8+ T cells. The study suggests that the distinct microbiome and metabolomic features in NAFLD-HCC have the ability to modulate peripheral immune responses (Behary et al., 2021). Nevertheless, more comprehensive investigation is required to acquire a deeper understanding of the changes in T cell phenotype induced by metabolites released by gut microbiota in HBV-related diseases.

## 7 Discussion

Emerging evidence suggests that the gut microbiota plays a significant role in immune regulation and has a notable impact on disease progression and treatment outcomes in the context of chronic HBV infection (Yun et al., 2019; Zeng et al., 2020; Shen et al., 2023). The effects of specific gut microbiota, metabolites derived from the gut microbiota, and immune cells on the liver-gut microbiota interaction in HBV-CLD have been systematically and comprehensively described. Significant dysbiosis of the gut microbiota has been observed in HBV-CLD (Wang et al., 2017). Dysbiosis-induced gut permeability enables the translocation of microbial products into the liver, triggering immune activation, inflammation, and immune system dysregulation, thereby exacerbating liver inflammation in chronic HBV infection.

Cross-sectional correlation studies have initially provided preliminary descriptions of changes in the abundance of different microbiota in CHB (Chen et al., 2011b, 2020; Xu et al., 2012; Wei et al., 2013; Leclercq et al., 2014; Wang et al., 2017, 2020; La Rosa et al., 2019; Pedersen et al., 2019; Yun et al., 2019; Shen et al., 2023). However, the alterations in the gut microbiota community under the circumstance of HBV infection, certain limitations still remain. The application of 16S rRNA sequencing can only gain species annotation information to the extent of genus level. Consequently, a wealth of information at the species level remains untapped. Moreover, most of these findings still require further validation through cohort data. Longitudinal cohort data will offer more extensive, stable, and reliable insights into the dynamics of the microbiota during the progression of HBV-CLD and the impact of antiviral therapy on microbiota structure. Establishing a causal relationship between gut dysbiosis and immune dysregulation in CHB infection remains challenging.

Combining multi-omics data analysis allows for capturing more comprehensive information on the “Gut-liver” axis. Humanized aseptic animal models have further advanced the exploration and validation of the mechanisms underlying the relationship between gut microbiota and metabolic data in HBV-CLD patients. In addition to overall changes in the structure of intestinal bacteria and deviations, some specific flora has been identified as pathogenic factors. Cytolysin-producing *Enterococcus faecalis*, *Klebsiella pneumoniae*, and *Enterococcus gallinarum*, for example, have been associated with alcoholic liver disease (Duan et al., 2019), NASH (Yuan et al., 2019), and autoimmune hepatitis (Bogdanos and Sakkas, 2018), respectively. These groundbreaking findings have accelerated the development of novel gut microbiota-based therapies for liver diseases. Probiotics and prebiotics, which aim to restore the dysbiosis and promote the growth of beneficial bacteria, have shown beneficial effects in reducing liver inflammation and viral load. FMT has also shown preliminary success in improving liver function and immune regulation in chronic viral hepatitis.

Numerous challenges remain to be addressed in future. For example, changes in the gut microbiota during the course of HBV infection give rise to alterations in gut metabolites that affect the replication and clearance of HBV. It’s essential to investigate whether the supplementation of certain gut microbiota metabolites, such as SCFAs, BAs, choline, or the adoption of a specific diet, could directly contribute to an antiviral effect against HBV. Furthermore, for common methods of gut microbiota intervention, such as total-FMT, if a synergistic antiviral effect is observed, it’s necessary to measure the changes in the content of gut metabolites brought about by FMT. Additionally, it’s important to analyze the shifts in the types of gut microbiota in individuals with HBV infection after total-FMT, with a focus on identifying species that exhibit a significant increase or decrease in abundance. This analysis might potentially offer insights into the relationship between specific bacterial species and the clearance of HBV infection, as well as whether these bacterial species shifts are associated with alterations in the immune status of individuals infected with HBV. Additionally, the efficacy of FMT in different subject is variable. The differences in the original host’s gut microbiota composition and immune environment need to be elucidated. Further investigation is still required to explore the changes in the gut and immune environments following FMT and their relationship with the replication and clearance of HBV.

## Author contributions

S-QL: Validation, Visualization, Writing – original draft, Writing – review & editing, Investigation. YS: Writing – original draft, Writing – review & editing, Investigation, Validation, Visualization. JZ: Validation, Visualization, Writing – review & editing. C-ZW: Validation, Visualization, Writing – review & editing. S-DW: Validation, Visualization, Writing – review & editing. WJ: Conceptualization, Funding acquisition, Validation, Writing – review & editing.
